# RNA based mNGS approach identifies a novel human coronavirus from two individual pneumonia cases in 2019 Wuhan outbreak

**DOI:** 10.1080/22221751.2020.1725399

**Published:** 2020-02-05

**Authors:** Liangjun Chen, Weiyong Liu, Qi Zhang, Ke Xu, Guangming Ye, Weichen Wu, Ziyong Sun, Fang Liu, Kailang Wu, Bo Zhong, Yi Mei, Wenxia Zhang, Yu Chen, Yirong Li, Mang Shi, Ke Lan, Yingle Liu

**Affiliations:** a State Key Laboratory of Virology, Modern Virology Research Center, College of Life Sciences, Wuhan University, Wuhan, People’s Republic of China; b Department of Laboratory Medicine, Zhongnan Hospital of Wuhan University, Wuhan, People’s Republic of China; c Department of Laboratory Medicine, Tongji Hospital, Tongji Medical College, Huazhong University of Science and Technology, Wuhan, People’s Republic of China; d School of Medicine, Sun yat-Sen University, Guangzhou, People’s Republic of China

**Keywords:** 2019-nCoV, Wuhan pneumonia, metagenomic next-generation sequencing, phylogenetic analyses, virus evolution

## Abstract

From December 2019, an outbreak of unusual pneumonia was reported in Wuhan with many cases linked to Huanan Seafood Market that sells seafood as well as live exotic animals. We investigated two patients who developed acute respiratory syndromes after independent contact history with this market. The two patients shared common clinical features including fever, cough, and multiple ground-glass opacities in the bilateral lung field with patchy infiltration. Here, we highlight the use of a low-input metagenomic next-generation sequencing (mNGS) approach on RNA extracted from bronchoalveolar lavage fluid (BALF). It rapidly identified a novel coronavirus (named 2019-nCoV according to World Health Organization announcement) which was the sole pathogens in the sample with very high abundance level (1.5% and 0.62% of total RNA sequenced). The entire viral genome is 29,881 nt in length (GenBank MN988668 and MN988669, Sequence Read Archive database Bioproject accession PRJNA601736) and is classified into β-coronavirus genus. Phylogenetic analysis indicates that 2019-nCoV is close to coronaviruses (CoVs) circulating in Rhinolophus (Horseshoe bats), such as 98.7% nucleotide identity to partial RdRp gene of bat coronavirus strain BtCoV/4991 (GenBank KP876546, 370 nt sequence of RdRp and lack of other genome sequence) and 87.9% nucleotide identity to bat coronavirus strain bat-SL-CoVZC45 and bat-SL-CoVZXC21. Evolutionary analysis based on ORF1a/1b, S, and N genes also suggests 2019-nCoV is more likely a novel CoV independently introduced from animals to humans.

## Introduction

The epidemic of emerging infectious diseases worldwide poses a great threat to public health. It is noted that most epidemic is caused by viral cross-species transmission from animals to human. Surveillance by fast and accurate diagnostic methods is crucial for the disease control and patient treatment. Thanks to the development of metagenomic next-generation sequencing (mNGS) methodology, the direct investigation of infectious microorganism from original clinical samples is currently achievable [1]. Particularly, RNA based mNGS approach could simultaneously reveal the entire “infectome” (i.e. RNA viruses, DNA viruses, bacteria and eukaryotes) present within an organism, because all except for prion express RNA [2]. Furthermore, RNA sequencing goes beyond pathogen identification to reveal relevant data on pathogen abundance, genome sequence, and gene expression, providing important insight into the cause of disease such that it represents an avant-guard diagnostic tool in the information age. Recently, in December 2019, an outbreak of unusual pneumonia caused by unknown infection was reported in Wuhan, China [3]. The earlier cases (before January) were all linked to Huanan Seafood Market in Wuhan before the disease was further spread to other cities of China and even overseas. Since this is an outbreak with unknown etiology, we report the use of RNA based mNGS approach for a rapid identification and characterization of a potential pathogen, which is therefore of great importance for disease control and prevention.

## Materials and methods

### Ethics statement

This study was approved by the Ethics Committee of the Zhongnan Hospital of Wuhan University. The mNGS analyses of BALF samples were performed on existing samples collected during standard diagnostic tests, posing no extra burden to patients.

### Sequence of events

**2nd January 2020.** Obtained BALF samples from two patients with unusual pneumonia.

**3rd January 2020.** Performed SARS-specific RT-PCR assay, yielded partial RdRp fragment, and revealed potential pathogen.

**4th January 2020.** Extended RdRp fragments and obtained more genome fragments, and started mNGS RNA library preparation

**5th January 2020.** Completed mNGS RNA library preparation.

**6th January 2020.** Started mNGS sequencing on Miseq platform.

**7th January 2020.** Received sequencing data, started pathogen identification pipeline, obtained virus genome, corrected the genome end with mapping, identified 2019-nCoV as sole pathogen, and the final CoV genome was 29,881 nt.

**8th January 2020.** Performed genome comparisons and evolutionary analyses.

Since 3rd January 2020, instant progress reports have been sent to Chinese Center for Disease Control and Prevention (CDC), keeping pace with every advancement we made in pathogen identification and characterization.

### Library preparation and sequencing

Total RNA extracted from BALF samples (collected on 2nd January 2020) were subject to metagenomic next-generation sequencing (mNGS) testing. The concentration of RNA samples were low (<0.5 ng/ul) based on measurement by Qubit RNA HS Assay Kit (Thermo Fisher Scientific), and therefore the library preparation was performed with Trio RNA-Seq kit (NuGEN Technologies, USA) which targeted low concentration RNA samples and contained AnyDeplete probe that removes human ribosomal RNA. The resulting libraries were subject to 150 bp pair-end sequencing with an Illumina Miseq platform. The sequencing results were obtained in less than 24 h.

### Pathogen discovery and characterization

To identify potential pathogens from the mNGS sequencing results, a pathogen discovery pipeline was carried out on sequenced data. Briefly, reads containing adaptor sequences and low-complex regions were removed from the dataset. Human reads were also removed by mapping against the reference human genome. All non-human and non-repeat sequence reads were then compared to a reference virus database (downloaded from https://ftp.ncbi.nih.gov/blast/db/ref_viruses_rep_genomes.tar.gz) and the non-redundant protein database (nr) using blastn and diamond blastx programs [4], respectively. Taxonomy lineage information was obtained for each blast hits by matching the accession number with the taxonomy database, which was subsequently used to identify reads of virus origin. Bacterial pathogen identification was carried out by using the Metaphlan2 program [5].

Reads were also assembled de novo using Megahit [6], with the virus genome identified based on the blast procedure described above. To validate the assembled genome sequences, reads were subsequently mapped to the genomes and a majority consensus sequences were determined for each sample. Minor variation calling was performed after mapping using Genious software package, with a minimum coverage set to 20 and minimum variant frequency set to 0.05. In addition to mapping, the virus genomes were also confirmed with Sanger sequencing using primers designed based on the NGS sequences.

### Phylogenetic and recombination analyses

Reference sequences associated with CoVs were downloaded from GenBank and aligned using mafft program. Phylogenetic trees (both amino acid and nucleotide alignment) were reconstructed using the maximum likelihood method in PhyML 3.0 [7], employing a best fit substitution model and a SPR branch swapping algorithm. Recombination event were discovered from phylogenetic analyses and confirmed with similarity plot implemented in the Simplot program [8].

## Results and discussion

On 2nd January 2020, samples were collected from two unusual pneumonia patients from Zhongnan Hospital of Wuhan University. Patient 1 was a 39-year-old male staff at Huanan Seafood Market who experienced fever (up to 37.7°C) and aggravated cough with frothy white sputum for 5 days before admitted to the hospital on 25th December 2019. Patient 2 was a 21-year-old female who developed an intermittent febrile cough, chills, fever (up to 40°C), and frothy white sputum after having a contact with Huanan Seafood Market staff on 22nd December 2019. She was admitted on 28th December after unsuccessful outpatient treatment. The results of clinical laboratory test on the first day of hospitalization are listed in Table 1. Chest CT scan of both patients showed patchy pulmonary opacities below the pleura in the bilateral lung field (Figure S1), which suggests viral infections may occur in both lungs. However, the subsequent routine anti-viral and anti-infection treatment did not alleviate their symptoms. On 31st December 2019, patient 1 had more severe symptoms, including poor mental states, shortness of breath, and 86% SpO2 without oxygen inhalation. A CT re-examination showed mild pleural effusion in the left lung, an increase in the density of ground-glass opacities, and an extension of the patchy area. The patient later experienced Type I respiratory failure on the same day. On 2nd January 2020, both patients were transferred to Wuhan Infectious Diseases Hospital for continuing treatment. To the date this manuscript was prepared, patient 1 and patient 2 were later discharged from the hospital in stable condition on 12th January and 11th January 2020, respectively.

**Table 1.** Clinical laboratory test on the first day of hospitalization.

Items	Case 1	Case 2	Normal range of lab test
WBC, ×10^9^/L	5.23	2.89	3.5–9.5
Neutrophils, ×10^9^/L/L	3.58	1.92	1.8–6.3
T lymphocyte, ×10^9^/L	1.32	0.46	1.1–3.2
Hb, g/L	138.6	127.5	115–150
Platelet, ×10^9^/L	170	117	125–350
Albumin, g/L	65.9	47	40–55
AST, U/L	92	33	7–45
ALT, U/L	30	30	13–45
CK, U/L	36	35	<171
CK-MB, U/L	11	10	0–25
LDH, U/L	313	247	110–245
UREA, mmol/L	2.81	2.7	2.8–7.60
CREA, μmol/L	73.9	57.2	49–90

Definition of abbreviations: ALT = alanine aminotransferase; AST = aspartate aminotransferase; CK = creatine kinase; CK-MB = creatinine kinase–MB isoenzyme; CREA = creatinine; UREA = Urea nitrogen; Hb = haemoglobin; LDH = lactate dehydrogenase; WBC = white blood count.

**Figure 1.**
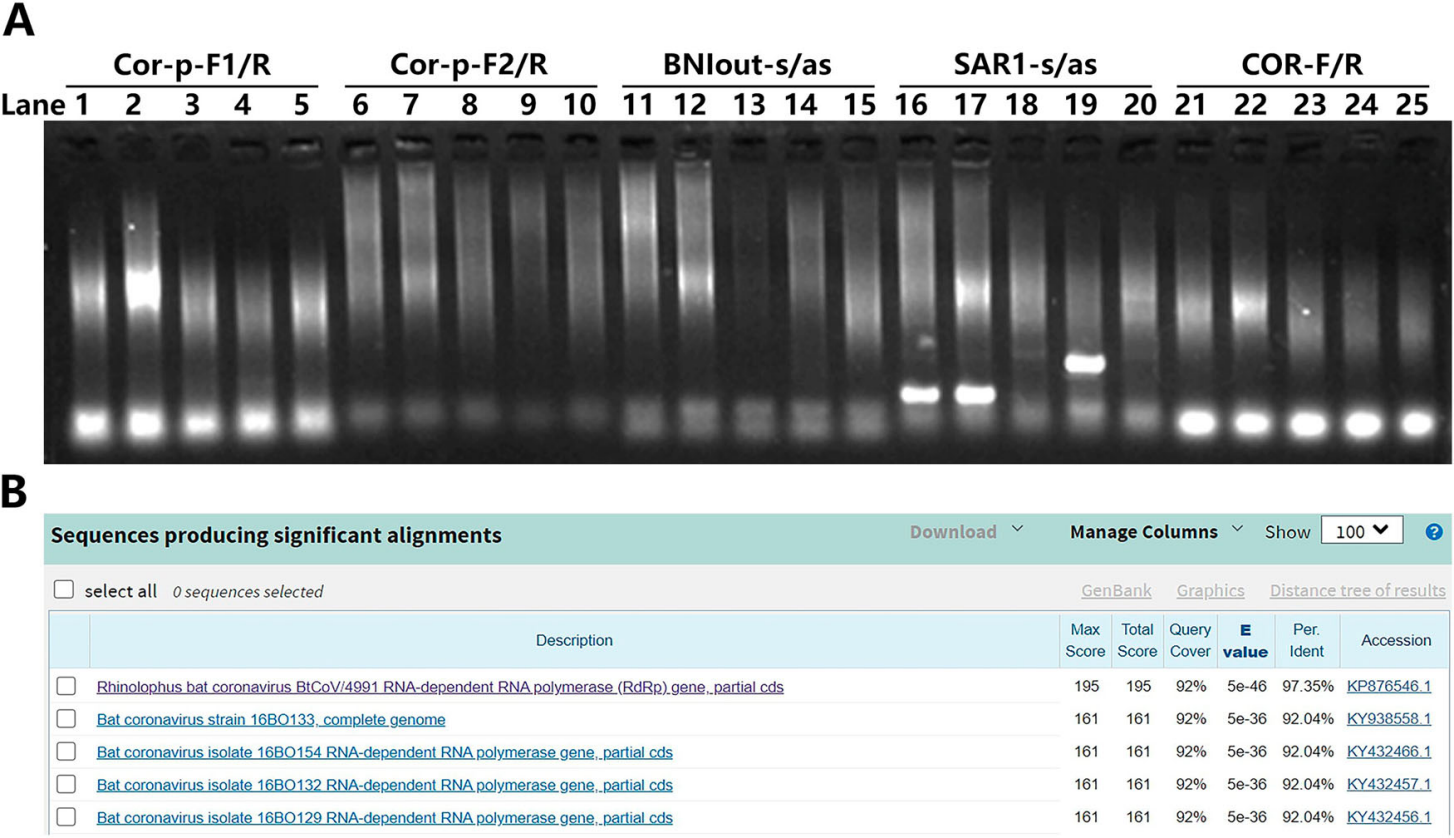
1st-round of RT-PCR assay, amplification and sequence analysis of unusual pneumonia outbreak in Wuhan. (A) RNA samples were subjected to SARS-CoV specific RT-PCR primer sets as indicated, only the SAR1-s/as set showed obvious band. Lane 1, 6, 11, 16, 21 are samples of patient 1. Lane 2, 7, 12, 17, 22 are samples of patient 2. Other lanes are samples of other patients who are irrelevant to this study. (B) The Blast result of PCR products of patient 1 and 2.

On 3rd January 2020, respiratory and blood samples obtained from the patients were subjected to routine clinical laboratory tests for respiratory pathogens, including Influenza virus, Respiratory syncytial virus, Adenovirus, Metapneumovirus, Mycoplasma pneumonia, Chlamydophila pneumonia, and Legionella, all yielding negative results. The remaining RNA samples were first subjected to SARS-CoV specific RT-PCR assays recommended by World Health Organization (WHO). However, only one set yielded positive results (Figure 1A). Further sequencing of the corresponding PCR product surprisingly suggested that the virus discovered is more closely related to BtCoV/4991 (97.35%) but not SARS-CoV (Figure 1B).

**Figure 2.**
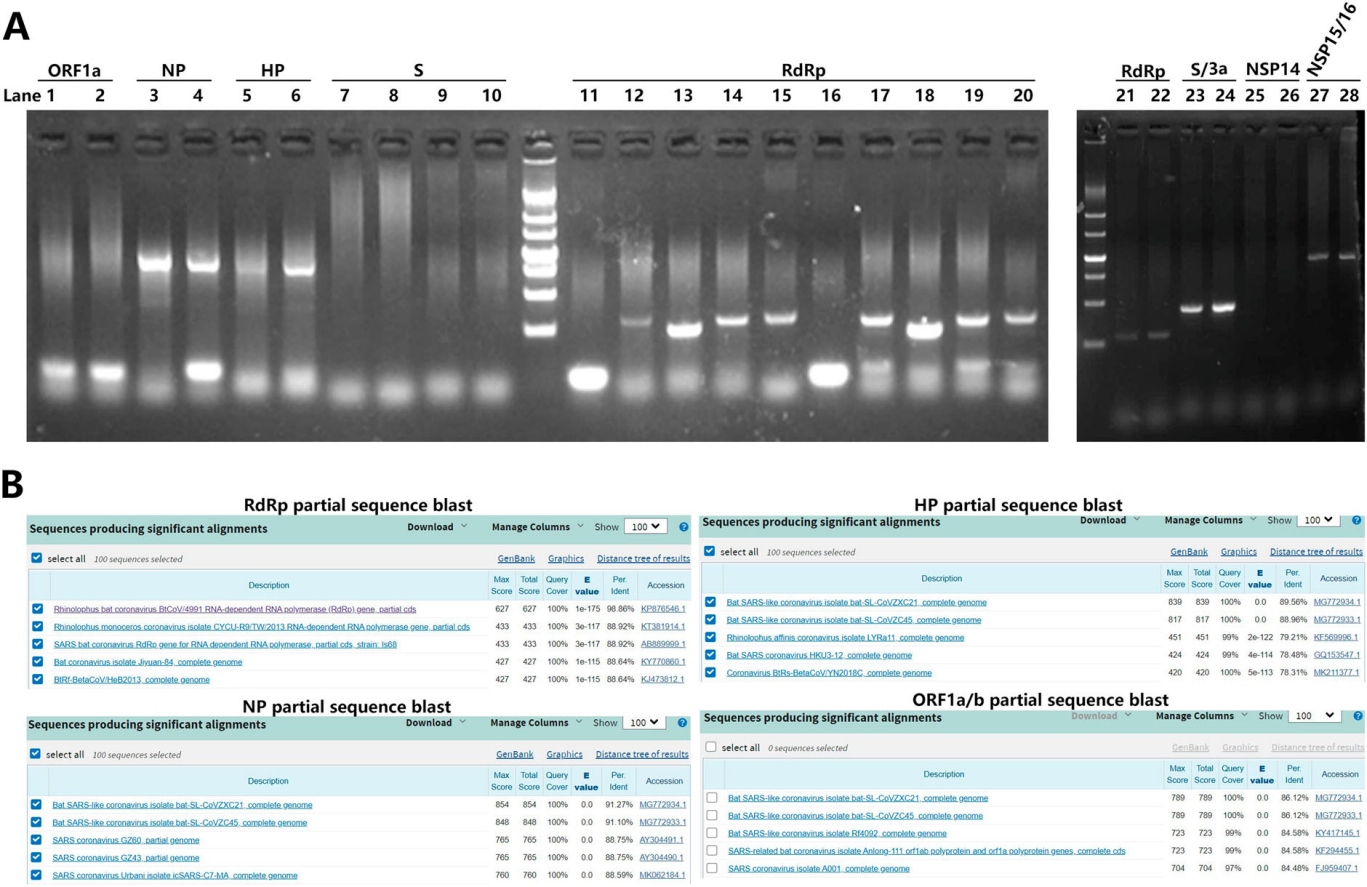
2nd-round of identification of unusual pneumonia. (A) RNA samples were subjected to multiple primer sets for different genes as indicated. Lane 1, 3, 5, 7, 9, 11, 12, 13, 14, 15, 21, 23, 25, 27 are samples of patient 1. Lane 2, 4, 6, 8, 10, 16, 17, 18, 19, 20, 22, 24, 26, 28 are samples of patient 2. (B) The PCR product of patient 1 and 2 were sequenced and the Blast result is shown.

On 4th January 2020, in 2nd-round RT-PCR assay, extended RdRp fragments and more genome fragments were identified, amplified, sequenced and analysed using new set of primers that were designed based on the 1st-round Blast analysis (Figure 2). These data further suggest that the pathogen of unusual pneumonia might be a coronavirus but not SARS-CoV. Meanwhile, total RNA extracted from BALF samples (collected on 2nd January 2020) were subject to metagenomic next-generation sequencing (mNGS) library construction.

On 5th January 2020, the mNGS library construction was completed.

On 6th January 2020, the resulting libraries were subject to 150 bp pair-end sequencing with an Illumina Miseq platform.

On 7th January 2020, the sequencing results were obtained in less than 24 h, with 7,369,020 and 4,522,558 reads generated for the samples of patient 1 and 2, respectively. To identify potential pathogens from the mNGS sequencing results, a pathogen discovery pipeline based on individual reads was carried out on sequenced data. Aside from those belonged to PhiX genome (in-library control), a majority of the viral reads (99.9% and 99.7% respectively for sample 1 and 2) were associated with coronaviruses. The raw sequence data minus human genomic information was uploaded to Sequence Read Archive (SRA) database (Bioproject accession PRJNA601736). On the other hand, bacterial pathogen identification was carried out by using the Metaphlan2 program, which revealed Capnocytophaga sp and Veillonella sp in sample 2 and none in sample 1, and both bacteria identified were not known for their pathogenicity. Collectively, coronavirus is likely to be the main microbial pathogen within these samples. The reads were assembled de novo using Megahit to form a ∼30 kb contigs with sequence homology to CoV. After confirmation with read mapping, the final CoV genome was 29,881 nt.

On 8th January 2020, the genome comparisons and evolutionary analyses were performed. Although some single nucleotide polymorphism (SNP) profiles were identified in the mNGS data (Table 2), the consensus genome sequences obtained from the patient 1 and 2 were identical (GenBank MN988668 and MN988669, respectively). These results indicated that these two individual patients were infected by the same CoV at separate times. We named the two clinical isolates as 2019-nCoV strain WHU01 and WHU02, respectively, according to WHO announcement. Based on the results of genome mapping, our data revealed extremely high viral abundance within the samples: the average genome coverage was 523.6X and 133.7X and the estimated abundance level were 1.5% and 0.62% of total reads sequenced for patient 1 and 2, respectively, suggesting active coronaviral replication in the lungs of both patients.

**Table 2.** Minor nucleotide variant identified from WHU01 and WHU02 genomes.

Strain	Region	Variant	Start Poisiton	End Position	Length	Change	Coverage	Polymorphism Type	Variant Frequency (%)	*P*-value
WHU01	1a	T	221	221	1	C → T	27	SNP (transition)	14.80	6.70E-07
WHU01	1a	A	1103	1103	1	T → A	119	SNP (transversion)	5.00	5.40E-14
WHU01	1a	A	1820	1820	1	G → A	97	SNP (transition)	11.30	2.00E-27
WHU01	1a	G	3916	3916	1	A → G	113	SNP (transition)	5.30	3.90E-14
WHU01	1a	TT	3919	3920	2	AA → TT	110	Substitution	5.50	1.30E-13
WHU01	1a	T	3923	3923	1	C → T	108	SNP (transition)	5.60	3.00E-14
WHU01	1a	T	5701	5701	1	C → T	247	SNP (transition)	5.30	5.70E-29
WHU01	1a	G	8892	8892	1	A → G	69	SNP (transition)	5.80	5.40E-10
WHU01	1a	A	8895	8895	1	T → A	65	SNP (transversion)	6.20	4.20E-10
WHU01	1a	G	8975	8975	1	A → G	59	SNP (transition)	5.10	6.50E-08
WHU01	1a	C	9114	9114	1	T → C	43	SNP (transition)	7.00	7.70E-07
WHU01	1a		11,081	11,081	1	(T)8 → (T)7	78	Deletion (tandem repeat)	12.80	1.20E-20
WHU01	1a	C	13,074	13,074	1	T → C	110	SNP (transition)	5.50	3.30E-14
WHU01	1a	TT	13,282	13,283	2	AA → TT	78 → 79	Substitution	5.10	9.40E-10
WHU01	1b	A	15,079	15,079	1	C → A	57	SNP (transversion)	8.80	1.30E-13
WHU01	1b	T	18,252	18,252	1	A → T	192	SNP (transversion)	6.30	5.50E-23
WHU01	1b	T	19,163	19,163	1	C → → T	89	SNP (transition)	19.10	1.90E-47
WHU01	1b	A	20,234	20,234	1	C → A	67	SNP (transversion)	6.00	1.20E-09
WHU01	S	A	22,315	22,315	1	G → A	182	SNP (transition)	6.60	4.70E-28
WHU01	S	A	22,447	22,447	1	C → A	54	SNP (transversion)	5.60	2.00E-07
WHU01	S	C	24,322	24,322	1	A → C	325	SNP (transversion)	38.50	0
WHU01	Other ORF	A	26,313	26,313	1	G → A	29	SNP (transition)	10.30	1.50E-08
WHU02	1a	T	1100	1100	1	C → T	390	SNP (transition)	6.70	1.50E-56
WHU02	1a	A	1103	1103	1	T → A	391	SNP (transversion)	5.90	3.10E-51
WHU02	1a	A	1820	1820	1	G → A	382	SNP (transition)	5.20	1.00E-41
WHU02	1a	C	6823	6822	0	+C	129	Insertion	5.40	2.50E-16
WHU02	1a	A	10,778	10,778	1	T → A	323	SNP (transversion)	5.30	2.40E-32
WHU02	1a	T	11,366	11,366	1	A → T	250	SNP (transversion)	6.00	4.40E-31
WHU02	1a	T	11,562	11,562	1	C → T	397	SNP (transition)	13.60	1.30E-138
WHU02	1b	T	13,692	13,692	1	A → T	356	SNP (transversion)	7.00	1.60E-57
WHU02	1b	C	14,306	14,306	1	T → C	279	SNP (transition)	7.90	9.20E-50
WHU02	1b	A	14,315	14,315	1	G → A	244	SNP (transition)	10.70	6.90E-57
WHU02	Other ORF	A	26,504	26,504	1	G → A	63	SNP (transition)	6.30	1.50E-10

Since 3rd January 2020, instant progress reports have been sent to the Chinese Center for Disease Control and Prevention (CDC), keeping pace with every advancement we made in pathogen identification and characterization.

**Figure 3.**
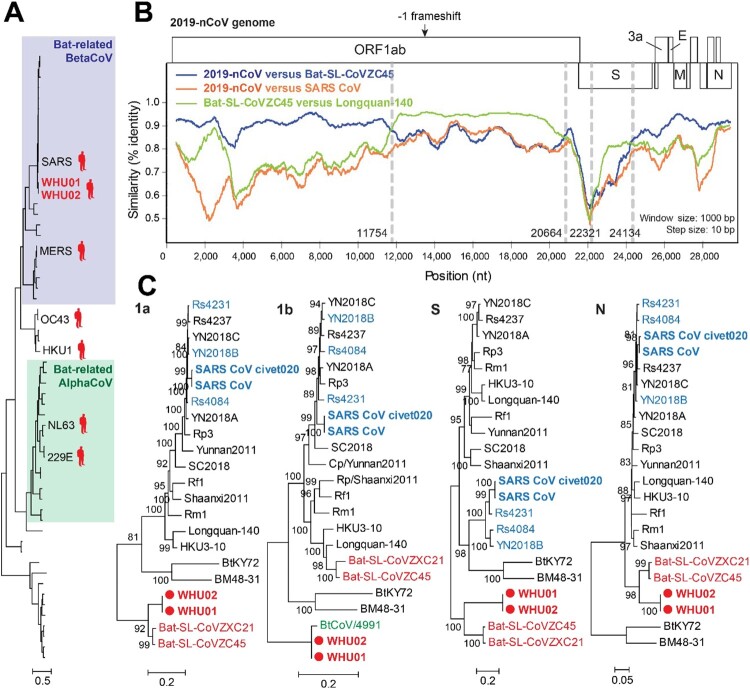
Origin and evolutionary history of newly identified CoVs. A. the position of 2019-nCoV in the context of all reference CoVs. The phylogeny is constructed based on ORF1b protein alignment. For clarity, names were only shown for human-associated viruses. Bat associated diversity is shaded with blue and green boxes for alpha- and beta-CoVs respectively. B. genome structure of newly identified viruses and its sequence similarity against bat-SL-CoVZC45 and SARS-CoV in a 1000bp sliding window across the entire genome. Recombination breakpoints are shown as dashed vertical lines. C. the relationship of WHU viruses with the other SARS-like CoVs. Phylogeny is reconstructed based on the nucleotide sequence of four genes: namely 1a, 1b, S, and N. Those grouped with WHU at S gene are marked red, and those grouped with SARS CoVs at S gene are marked blue.

The genomes of the 2019-nCoV were further analysed to determine its origin and evolutionary history. Full genome comparisons indicated that 2019-nCoV is close to CoVs circulating in Rhinolophus (Horseshoe bats). For example, it shared 98.7% nucleotide identity to bat coronavirus strain BtCoV/4991 (GenBank KP876546, only 370 nt sequence of RdRp gene) and 87.9% nucleotide identity to bat CoV strain bat-SL-CoVZC45 and bat-SL-CoVZXC21, indicating that it was quite divergent from the currently known human CoV, including SARS-CoV (79.7%). To put 2019-nCoV in the context of whole *Coronaviridae* family, we aligned ORF1b protein sequences from representative CoVs diversity for phylogenetic analyses (Figure 3A). It revealed that the 2019-nCoV is grouped under genus β-coronavirus, subgenus Sarbecovirus, and a cluster that is known to harbour bat-SL-CoVs, many of which were associated with *Rhinolophus sp.* (horseshoe bats).

To reveal a more detailed relationship between 2019-nCoV and other CoVs, we reconstructed phylogenies based on nucleotide alignment of key viral genes, including ORF1a/b, S, and N. Within this cluster, the 2019-nCoV also shared close relationship with CoVs originated from Rhinolophus bat. For ORF1b gene, the closest relative is BtCoV/4991 (KP876546, 98.65% nucleotide identity, based on partial RdRp gene comparisons) identified from *Rhinolophus affinis* from Yunnan; whereas for the rest of the genes analysed, the closest are bat-SL-CoVZXC21 (76.5–91.2% nucleotide identity) and bat-SL-CoVZC45 (76.9–91.2% nucleotide identity) identified from *Rhinolophus sinicus*. The close relationship with BtCoV/4991 is quite essential in tracing the potential reservoir host of 2019-nCoV. Unfortunately, the BtCoV/4991 sequence was only partial (373bp in length) and thus no comparisons can be made for the rest of genomes. However, the presence of such close relatives in bat viruses strongly suggests that it might be originated from a recent and independent introduction from bats to humans, although its immediate hosts remain to be identified.

Through gene-specific phylogenetic analyses, we also identified phylogenetic incongruence for 2019-nCoV compared with other bat-SL-CoVs at different genes, suggesting potential recombination event. Specifically, 2019-nCoV was closely related to strains bat-SL-CoVZXC21 and bat-SL-CoVZC45 at ORF1a, S, and N genes, but not at ORF1b gene. At ORF1b gene, bat-SL-CoVZXC21 and bat-SL-CoVZC45 were related to strains Longquan-140 and HKU3-10 (Figure 2C). Simplot analyses based on genome alignment of 2019-nCoV, bat-SL-CoVZC45, Longquan-140, and SARS-CoV suggest that the recombinant strain was not likely to be 2019-nCoV, but bat-SL-CoVZC45 (Figure 3B). And it also revealed at least four recombination breakpoints at positions 11,754, 20,664, 22,321, and 24,134 nt of the genome alignment, respectively (Figure 3B).

In conclusion, we have identified a novel CoV from two patients with unusual pneumonia. Although the direct association with the disease is yet to be confirmed with more experimental data, our results provide several lines of evidence that the virus is most likely associated with this disease: (i) the viral titre is very high, with the abundance level reaching 1.5% and 0.62% of total reads sequenced, surpassing the highest expressed host genes to be one of the most dominant RNA molecules in the host transcriptome, an important sign that the virus is then under active replication [9]; (ii) since our RNA mNGS approach targets the total infectome (except for prion) [10], the fact that no other pathogens were identified from the infected sample underlines the unique role played by 2019-nCoV; (iii) the virus is grouped within the notorious CoV clade (i.e. SARS-like) with history of cross-virus transmission to humans [11] and has been demonstrated to have strong zoonotic potential [12]; and while this manuscript was under preparation, we noticed another case report from Wuhan which identified a same virus as the one found in this study [13]. Collectively, these results use the rich information present in the RNA metagenomics to evaluate potential pathogens, which highlights a future trend of viral diagnosis in the age of information.

## Supplementary Material

Supplemental Material
