# Supplementary material for: RNA based mNGS approach identifies a novel human coronavirus from two individual pneumonia cases in 2019 Wuhan outbreak
Source: Emerg Microbes Infect. 2020 Feb 5;9(1):313–9. doi: 10.1080/22221751.2020.1725399 (PMC7033720; doi:10.1080/22221751.2020.1725399)
Supplement: Supplemental Material [file TEMI_A_1725399_SM8296.docx]

Supplementary Material:

**Figure S1**


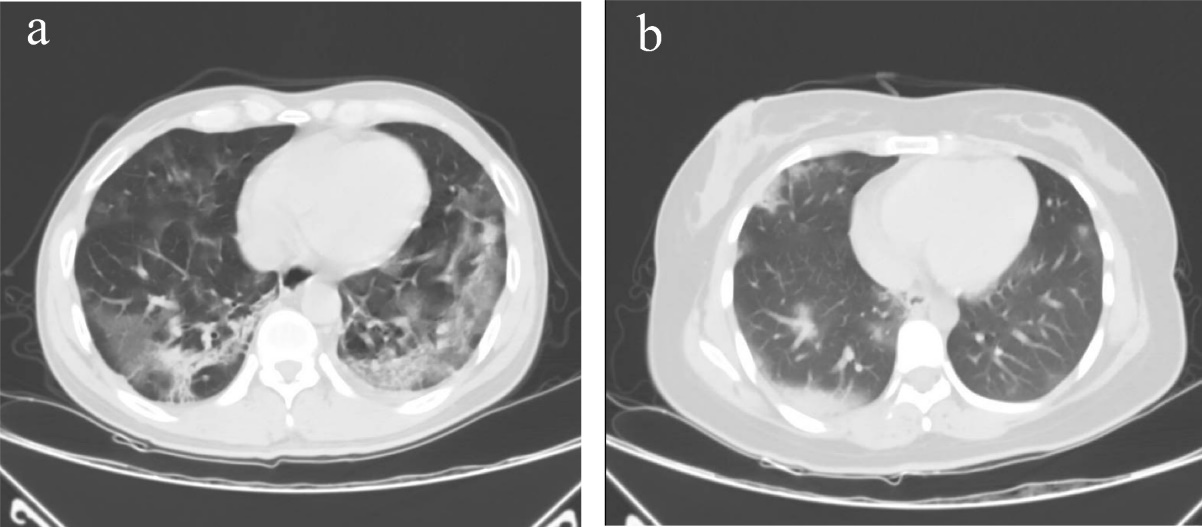


**Figure S1. Chest imaging of pulmonary window taken of the two confirmed novel CoV infection patients.** There was bilateral filtration in lower lobes. (a) The chest imaging of the first case was obtained on December 30, 2019. (b) The chest imaging of the second case was also obtained on December 30, 2019.
